# The global procurement landscape of leishmaniasis medicines

**DOI:** 10.1371/journal.pntd.0009181

**Published:** 2021-02-18

**Authors:** Hye Lynn Choi, Saurabh Jain, José A. Ruiz Postigo, Bettina Borisch, Daniel Argaw Dagne

**Affiliations:** 1 World Health Organization, Geneva, Switzerland; 2 Institute of Global Health, University of Geneva, Geneva, Switzerland; National Institute of Allergy and Infectious Diseases, UNITED STATES

## Abstract

Ensuring access to essential medicines for leishmaniasis control is challenging, as leishmaniasis is a very small and unattractive market for pharmaceutical industry. Furthermore, control programmes are severely underfunded. We conducted an analysis of global procurement of leishmaniasis medicines for the past 5 years in order to shed light on the current leishmaniasis market landscape and supply and demand dynamics. We estimated global demand of each leishmaniasis medicines, the amount of each medicine required to treat all reported cases, based on the number of cases reported to WHO and the first-line treatment regimen used in each country. Procurement data were obtained from procurement agencies, international organizations, WHO, national leishmaniasis programmes and manufacturers. Expert interviews were conducted to have a better understanding of how medicines were procured and used. The comparison of estimated need and procurement data indicated discrepancies in supply and demand at global level as well as in the most endemic countries. The extent of the gap in supply was up to 80% of the needs for one of the leishmaniasis medicines. Mismatch between supply and demand was much wider for cutaneous leishmaniasis than visceral leishmaniasis. This study presents a current picture of procurement patterns and imbalance in global supply and demand. Addressing improved access and supply barriers requires concerted and coordinated efforts at the global and national levels. Priority actions include setting up a procurement coordination mechanism among major procurers, partners and national programmes where forecasting and supply planning is jointly developed and communicated with manufacturers. In addition, continuous engagement of manufacturers and advocacy is critical to diversify the supplier base and ensure quality-assured and affordable generic medicines for leishmaniasis.

## Author summary

Even though more than 1 billion people are at the risk of leishmaniasis infection, one of the neglected tropical diseases, there is limited access to essential medicines for the treatment of leishmaniasis. We compared the procurement data collected from major procurers with the global needs estimated from the number of cases reported to WHO and the first-line treatment regimen used in each country for the past 5 years to better understand the market landscape and supply and demand dynamics. The results showed imbalances and mismatches in supply and demand at global and national levels. Improved coordination among national leishmaniasis control programmes, procurers, manufacturers and partners is needed to address access barriers. This should include proactive engagement of manufacturers to secure uninterrupted supply of quality-assured medicines, setting up the procurement coordination mechanism with major procurers for the development of joint forecasting and market shaping interventions, and promoting high-level advocacy.

## Introduction

Access to quality assured essential medical products and technologies is one of the building blocks of the WHO’s health systems framework [1]. Sustainable Development Goals 2030 highlight the need to expand access to essential medicines and health products through two targets, 3.8 and 3.b [2]. therefore, access to health products remains a key indicator for countries’ progress towards universal health coverage. This becomes more important for neglected tropical diseases (NTDs) which are proxy for poverty and disadvantage [3]. NTDs not only affect populations with low visibility and little political voices disproportionately but also cause stigma and discrimination and have an important impact on morbidity and mortality.

Leishmaniasis, one of the NTDs, is a significant public health problem affecting the world’s most vulnerable and poorest populations [4]. Of all tropical diseases, leishmaniasis arguably ranked second in mortality and seventh in loss of disability-adjusted life years [5]. Over 1 billion people are at risk of infection in high-burden countries [6]. It is estimated that there are 700 000–1.2 million new cases and 14 000–40 000 deaths from the disease each year worldwide [7,8]. There are three main clinical forms of the disease: visceral leishmaniasis (VL, also known as kala-azar), cutaneous leishmaniasis (CL) and mucocutaneous leishmaniasis [9]. In 2018, of the 200 countries and territories that reported to WHO, 97 (49%) were considered endemic for leishmaniasis. Of those 200, 88 (44%) were considered endemic for CL and 78 (39%) were considered endemic for VL. Of the 200, 69 (35%) were endemic for both CL and VL. [10]. Post kala-azar dermal leishmaniasis (PKDL) appears in a subset of patients treated for VL, usually 6 months to 1 or more years after cure of VL. PKDL is most common in Bangladesh, India and Sudan [11].

World Health Assembly resolution 60.13 on the control of leishmaniasis was adopted in 2007 [12], calling for people affected by leishmaniasis to have access to treatment of both VL and CL. This has become a global priority, and leishmaniasis has subsequently gained the attention of both the public health community and donors. WHO has set targets and milestones for the control of leishmaniasis: elimination of VL as a public health problem, defined as less than 1% case fatality rate globally due to primary VL by 2030 and treatment of 90% of CL cases by 2030 [13].

Resolution 60.13 also urges Member States to advocate quality-assured and affordable medicines, and appropriate national drug policies to control leishmaniasis [12]. However, treatment options are limited and access to quality-assured and affordable medicines for leishmaniasis has been a challenge. The medicines available for VL and CL are pentavalent antimonials (sodium stibogluconate [SSG] and meglumine antimoniate [MA]), paromomycin (PM), miltefosine (MF), pentamidine isethionate and amphotericin B (amphotericin B deoxycholate and liposomal amphotericin B [LAMB]).

Antimonials, despite their toxicity and variable efficacy, were used alone as the standard treatment over the past 60 years, until new treatments were developed and introduced. Recent advances for VL such as combination therapy of SSG and PM, oral formulation of MF and LAMB provide better safety profiles, shorter treatment durations and reduced risk of resistance emergence [14,15]. For example, the current WHO-recommended combination therapy of SSG and PM for 17 days as a first-line treatment regimen for VL in East Africa in 2010 [4] replaced SSG monotherapy for 30 days, and LAMB (which has a much better safety profile) is now used for complicated VL patients, pregnant women or VL in HIV-coinfected patients in East Africa [16,17]. Single-dose LAMB was shown to have high initial and long-term efficacy and a low rate of adverse reactions in India [18,19], and single-dose LAMB has been adopted as first-line treatment by national VL programmes in South-East Asia [20,21]. In other VL foci in the Region of the Americas, the first-line treatment is MA for 20 days. A range of intervention options are recommended for the treatment of CL according to the infecting species and geographical regions: local therapy in combination with intralesional antimonials and cryotherapy, thermotherapy, phototherapy, topical ointments with PM, systemic therapy of intramuscular or intravenous antimonials, MF, fluconazole and others [22]. PKDL patients are often treated with MF for 12 weeks and antimonials for 30–60 days in the Indian subcontinent and East Africa, respectively [23,24].

Despite the development of new treatment options in recent years, barriers to accessing these medicines are one of the most critical impediments in making progress towards control or elimination of leishmaniasis. Leishmaniasis or, in general, neglected tropical diseases, are not an attractive or profitable market for the pharmaceutical industry, as the market size is small or uncertain and target populations are the poorest of the poor living in remote areas in low and middle-income countries. The neglected tropical diseases, overshadowed for many years by the “big three” (HIV/AIDS, malaria and tuberculosis), receive extremely little research and development and attract scant resources [25]. All leishmaniasis medicines are sourced from in some cases single, and in others a small number of manufacturers. All these elements contribute to market failure: inadequate production capacity, lack of registration in countries, supply insecurity, frequent shortages or stock-outs and high prices [26–28]. For example, affordability and availability have been key obstacles to expanded access to MF, the only oral medicine approved for the treatment of leishmaniasis. The global availability of MF, initially developed by a public–private partnership (PPP) and now owned by Knight Therapeutics, depends on a single source, as no other quality-assured generic medicine is available on the market. The current price of MF is three times higher than that in the initial PPP agreement, which is not affordable for the majority of patients [29]. Challenges in access are further compounded by inefficient supply chains, unreliable forecasts, suboptimal distribution and delivery systems in countries, and complex registration processes [26,28]. Circulation of substandard and falsified leishmaniasis products poses a threat to patient safety and leads to treatment failures, parasite resistance and death [29–31].

An analysis of the past procurement of leishmaniasis medicines was conducted to better understand the market dynamics and procurement landscape. We aimed to provide some insights into the leishmaniasis market for manufacturers, partners, procurers and national leishmaniasis control programmes and the patterns in which selected countries are currently treating leishmaniasis through this procurement analysis.

## Methods

The global demand of each leishmaniasis medicine for VL, CL and PKDL was estimated based on the number of cases reported and the first-line treatment regimen used in each country. We used the number of cases of VL and CL including imported cases from the WHO Global Health Observatory data repository from 2013 to 2017 (Table 1). The number of PKDL cases was collected from leishmaniasis country profiles maintained and published by WHO and annual reports sent from national leishmaniasis control programmes in East Africa and on the Indian subcontinent where PKDL commonly occurs (Table 2).

**Table 1:** Number of VL and CL cases, by WHO region.

WHO region	Number of VL cases reported					Number of CL cases reported				
	2013	2014	2015	2016	2017	2013	2014	2015	2016	2017
AFR	4418	11 308	5924	6672	6130	7460	5770	8211	11 120	13 951
AMR	3395	3624	3459	3358	4422	50 176	51 509	46 118	49 430	49 678
EMR	4256	5140	4694	5144	5166	132 177	129 229	158 348	156 058	77 983
EUR	824	519	389	380	190	6620	5497	3964	4071	1112
SEAR	15 311	10 227	9279	6756	6174	173	1439	1283	2	6
WPR	122	295	515	322	192	5	1	2	14	8

AFR: African Region; AMR: Region of the Americas; EMR: Eastern Mediterranean Region; EUR: European Region; SEAR: South-East Asia Region; WPR: Western Pacific Region.

**Table 2:** Number of PKDL cases, by WHO region.

WHO region	Countries	2013	2014	2015	2016	2017
SEAR	India	499	421	648	1657	1982
SEAR	Bangladesh	325	318	239	154	102
SEAR	Nepal	No data	6	2	1	7
AFR	Ethiopia	8	20	11	14	20
AFR	Kenya	No data	3	5	No data	No data
AFR	Somalia	No data	No data	No data	No data	No data
AFR	Sudan	No data	52	43	87	72
AFR	South Sudan	56	53	54	No data	141
AFR	Uganda	No data	No data	No data	No data	No data

AFR: African Region; SEAR: South-East Asia Region.

Information on the first-line treatment regimen and the medicines used in each country was retrieved from WHO leishmaniasis country profiles and country treatment guidelines (Table 3). This was complemented by consultations with experts and officers responsible for national leishmaniasis control programmes in endemic countries. An average weight of 35 kg was used to calculate the number of units of medicine required in each regimen [4]. The age distribution of PKDL patients from the country profiles was used to estimate the number of patients who require MF 10 mg or 50 mg.

**Table 3:** First-line regimen used for VL, CL and PKDL, by country.

VL first-line treatment regimen selected	Use in countries
▪ LAMB 10 mg/kg as a single dose	SEAR (4 countries)
▪ LAMB 3–5mg/kg over 3 to 6 days up to total dose of 18–21 mg/kg	EUR (9 countries)
▪ Combination of pentavalent antimonials 20 mg (SB5+)/kg per day and PM 15 mg (11 mg base)/kg per day for 17 days	AFR and some EMR (8 countries)
▪ Pentavalent antimonials 20 mg SB5+/kg per day for 28 days (or 30 days)	AMR, EMR, EUR (43 countries)
**CL first-line treatment regimen selected**	
▪ Intralesional pentavalent antimonials 1−5 mL per session plus cryotherapy for 1–5 sessions (local therapy)	AFR, Pakistan, 80% of patients in EMR countries
▪ Pentavalent antimonials SB5+ 20 mg/kg per day intramuscularly or intravenously for 20 days (systemic therapy)	EUR, AMR, 20% of patients in EMR countries
**PKDL first-line treatment regimen**	
▪ MF 100 mg/day for 12 weeks (or 2.5mg/kg per day)	SEAR
▪ Pentavalent antimonials 20 mg/kg per day for 30–60 days	AFR

AFR: African Region; AMR: Region of the Americas; CL: cutaneous leishmaniasis; EMR: Eastern Mediterranean Region; EUR: European Region; LAMB: liposomal amphotericin B; MF: miltefosine; PM: paromomycin; SEAR: South-East Asia Region; VL: visceral leishmaniasis.

Procurement data were obtained from procurement agencies, international organizations, WHO, the Pan American Health Organization’ strategic fund, non-profit organizations and manufacturers who play a role in supplying leishmaniasis medicines. The quantity of each medicine delivered in the country from 2013 to 2017 (and presumably administered to the patients) was used for the analysis. We compared these numbers with the estimated quantity of medicines needed to treat the cases reported to WHO. We conducted interviews with experts in the field to have a better understanding of how medicines were procured for countries using domestic funds and through national tenders. The picture is not complete, but our attempt provides some insights into the leishmaniasis market landscape and the manner in which endemic countries are currently treating leishmaniasis cases.

## Results

**Fig 1:**
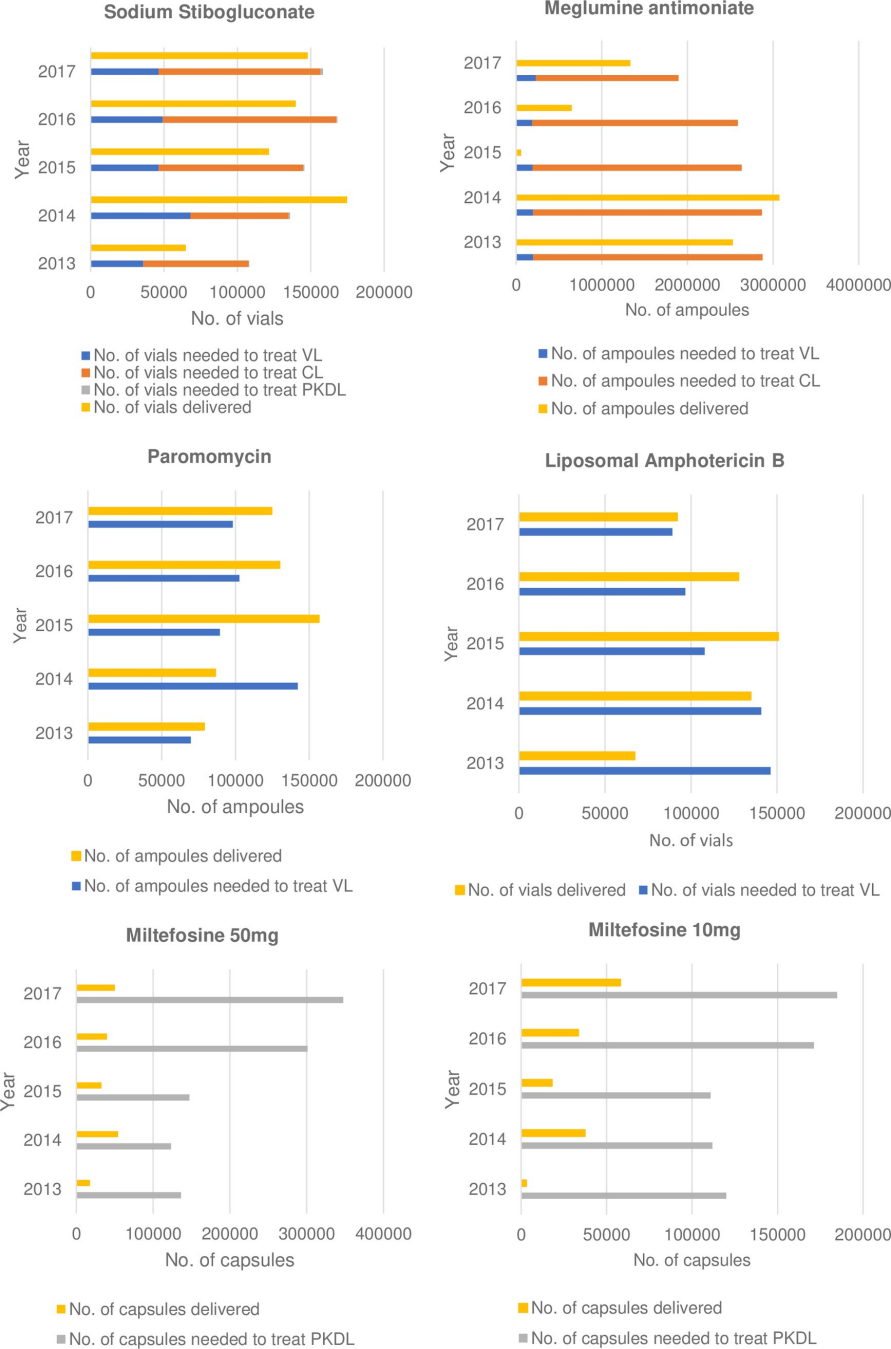
Global supply and demand of leishmaniasis medicines 2013–2017. (A) No. of liposomal amphotericin B vials needed to treat was corrected with number of patients who require liposomal amphotericin B as the second-line treatment to rescue severe and complicated cases in East Africa. (B) CL: cutaneous leishmaniasis; PKDL: post kala-azar dermal leishmaniasis; VL: visceral leishmaniasis.

Table 1 shows that there is no trend in the number of cases for VL and CL except decreasing numbers of VL in the Indian subcontinent, partly due to outbreaks of VL and CL in different countries. Data on volumes of leishmaniasis medicines procured by various institutions offer insights into how this unpredictable demand was met. The supply and demand data analysis from 2013 to 2017 for each medicine is presented in Fig 1.

Based on the estimated global demand of each medicine and the average unit cost, the total estimated size of the market for leishmaniasis medicines is worth of US$ 4 million to 7.1 million per year. MA and LAMB account for 74% of this global demand. The analysis indicates that global supply for SSG, MA and MF over 5 years did not meet the demand over 5 years by 10%, 40% and 73–79%, respectively, while PM was over-supplied by 15%. There was almost no gap in supply and demand for LAMB over the past 5 years. The LAMB was supplied to countries in the Indian subcontinent and East Africa through WHO and a Gilead donation agreement based on the annual forecast submitted by national programmes and stock monitoring supported by WHO.

**Fig 2:**
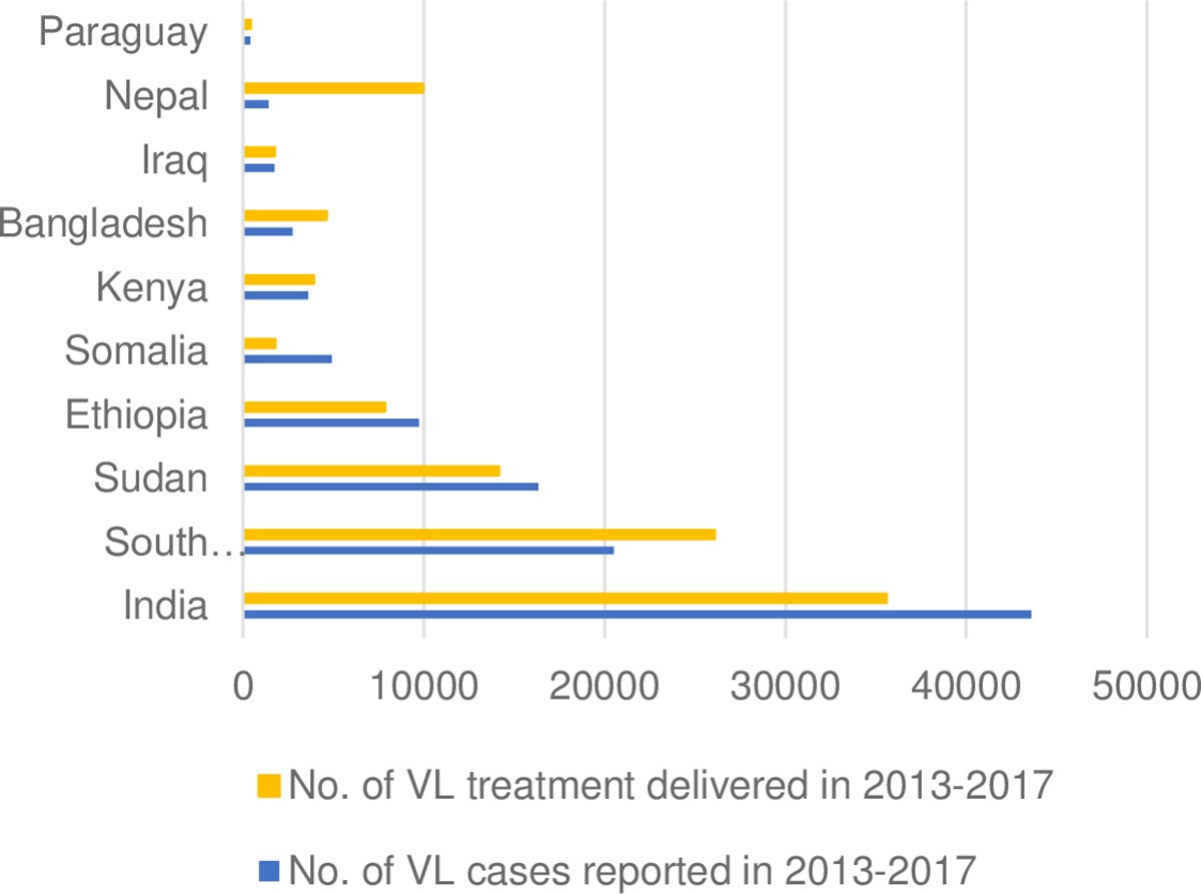
Supply and demand in top 10 VL countries, 2013–2017. (A) Antimonials are used to treat both VL and CL in Paraguay and Iraq. (B) We assumed that VL patients were treated with priority. (C) Where sodium stibogluconate and paromomycin is used in combination to treat VL, the number of treatments delivered is estimated based on the number of paromomycin vials procured and delivered, as sodium stibogluconate is also used to treat CL. (D) CL: cutaneous leishmaniasis; VL: visceral leishmaniasis.

Country-specific product procurement data were used to gauge use of medicines among patients in endemic countries and to compare the overall need for treatments based on the estimated number of leishmaniasis cases. Fig 2 presents the supply and demand dynamic from 2013 to 2017 cumulatively in the 10 countries with the highest number of VL cases reported, excluding Brazil, China and Morocco where no procurement data were available. Discrepancies go both ways, with an excess in the number of VL cases reported or treatments provided. Mismatches between the number of VL treatments delivered and the number of cases reported are apparent in India, South Sudan and Nepal. We learned from expert consultations that mismatch is possibly due to underreporting of cases, unexpected outbreaks, changes in treatment guidelines and overstock or stockout of products from previous years.

**Fig 3:**
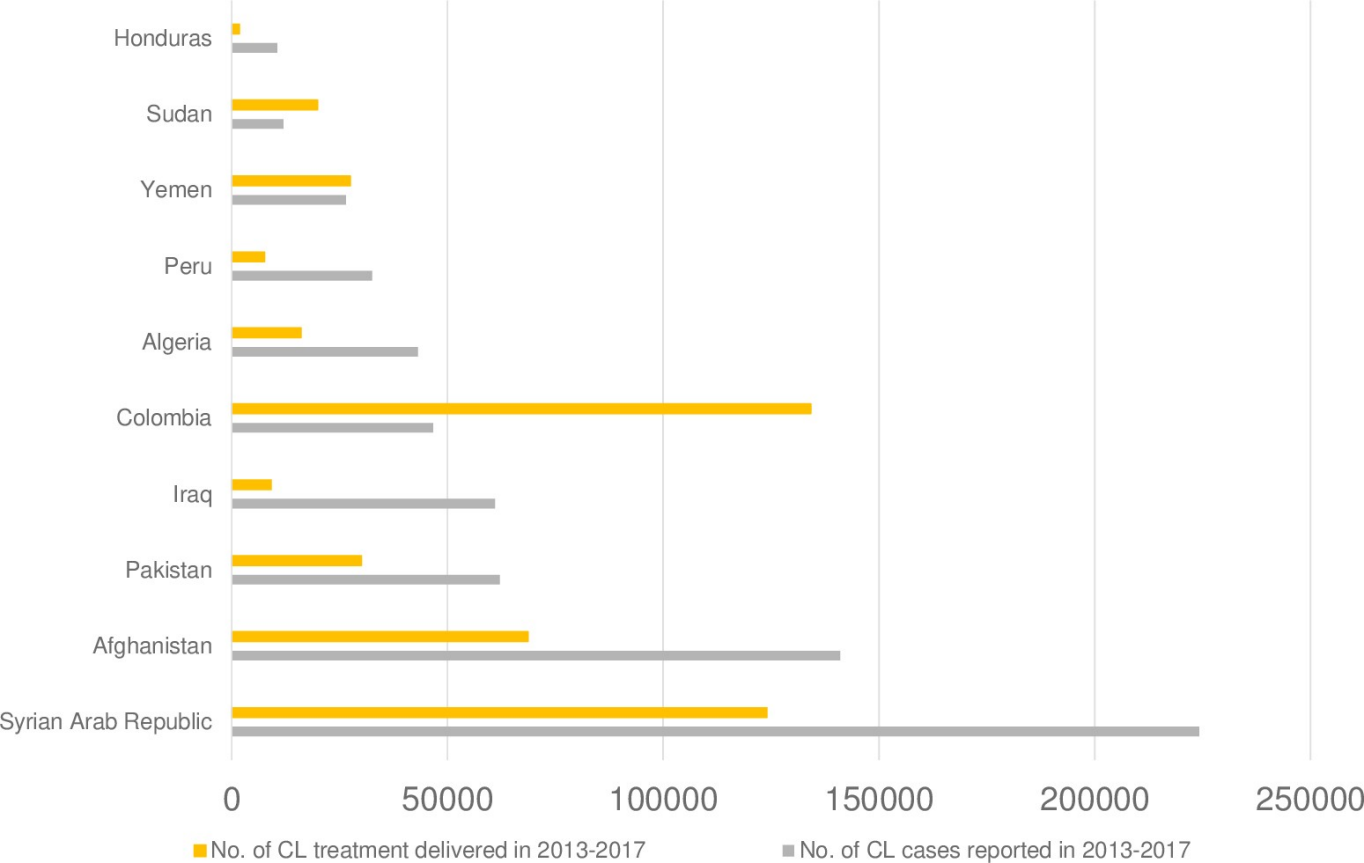
Supply and demand in top 10 CL countries, 2013–2017. CL: cutaneous leishmaniasis.

The supply and demand analysis in CL endemic countries from 2013 to 2017 cumulatively is presented in Fig 3. This includes the 10 countries with highest number of CL cases reported. Brazil and the Islamic Republic of Iran are not included in the analysis, as these two countries have procured antimonials using domestic funds and there was no procurement data available for this analysis. The experts highlighted that the large gap between supply and demand for CL in most of these countries may be associated with lack of funding for CL and political commitment. It is also due to frequent outbreaks of CL, which makes forecasting unreliable. Colombia procured more treatments than number of cases reported. An expert described the excess in cumulative supply in Colombia as possibly due to the drastic decrease in CL cases reported by the army engaged in operations against the guerrillas and narcotics traffickers when the peace agreement was signed in 2016.

## Discussion

The current market for leishmaniasis medicines is fragile and fragmented due to various factors such as lack of funding, little interest from the pharmaceutical industry and uncoordinated procurement with uncertain forecasting. This study attempted to better understand procurement practices and patterns in endemic countries in order to identify more effective market shaping strategies and to sustain manufacturers’ engagement.

An earlier study [26] expounded on barriers to access and availability of leishmaniasis products identified by various stakeholders. This analysis confirms that there is a significant supply and demand imbalance at the global and country levels. Possible reasons for such disparity include limited financing, supply disruption relying on single source, lack of coordination and collaboration among procurers, partners and manufacturers, unaffordable prices and an ineffective supply chain. It was also noted that some of the manufacturers (of MF and PM) do not keep the products in stock and require an entire batch to be purchased at once due to the small and unpredictable amount of orders they receive. This led to long delays or stockouts in case only small quantities are needed. Furthermore, not all leishmaniasis products are fully quality-assured. PM and SSG are under WHO Expert Review Panel (ERP) oversight, but not yet prequalified by the WHO Prequalification Programme or authorized by a stringent regulatory authority (SRA). There are also generic MF products used by governments that are neither reviewed by ERP, prequalified nor approved by SRA. In 2019, there were falsified MA ampoules circulating in the Islamic Republic of Iran and Pakistan [31].

Though this study focused on historical procurement analysis, anticipated treatment guideline changes and emerging potential resistance may further complicate future supply and demand dynamics. For example, demand for MF in this study was estimated based on the number of PKDL patients in the Indian subcontinent. The demand for MF is expected to increase in the future, as some countries such as Brazil are considering inclusion of MF as the first-line treatment for special groups over 50 years old for CL. New evidence of a combination regimen of LAMB and MF for people coinfected with HIV and VL in East Africa and South-East Asia and that of PM and MF for VL for East Africa may require updated treatment guidelines, which would then lead to an increase in MF need [32,33]. Demand for LAMB may also increase as countries such as Argentina, Brazil and Colombia are adopting the use of LAMB as the first-line treatment for VL. In the near future, evidence for combination regimens is expected for the treatment of PKDL as well, influencing the medicines demand considerably.

Our attempt to address the discordant supply and demand at global and country levels clearly calls for interventions and actions from all leishmaniasis stakeholders. A mechanism for coordination of quantification and supply planning is required to increase visibility of demand, have better negotiation power, meet minimum order quantity and help production planning for suppliers. Partners can also consider pooled procurement based on consolidated demand forecasting, either through a centralized or decentralized procedure, but in a coordinated manner such as using a call for orders. An in-depth feasibility study on pooled procurement is needed to inform the design of procurement models and procedures. Such a coordinating mechanism can also be a platform for market shaping and manufacturers’ engagement by sharing global forecast with manufacturers and organizing a joint price negotiation to achieve lower prices and better terms and conditions. Strategies for sustaining manufacturers’ interest or attracting potential, new suppliers are also needed, including promoting WHO prequalification or SRA approval. In order to facilitate such coordination among stakeholders, in 2016 WHO established the working group on access to medicines and diagnostics for leishmaniasis control to regularly discuss and address challenges related to procurement and donations with the major procurers. This working group can be one of the adequate platforms to implement proposed strategies and actions. Policy makers, leishmaniasis programme managers, donors, partners and in-country nongovernmental organizations should collaborate to strengthen national capacity for forecasting and supply chain management for leishmaniasis products and secure adequate funding for procurement. Accurate and updated forecasting together with efficient inventory and storage management are critical in order to avoid stock-outs and expiries in the countries, which then often require emergency procurement and prioritization of orders. Emergency stock established by the WHO leishmaniasis programme for 1000 patient treatments has been instrumental in responding to such needs and outbreaks in endemic countries. WHO can consider expanding the size of stock to be used as rotating stockpile, although this would require increased funding and resource mobilization.

This study is mainly limited by availability of procurement data analysed. Some of the differences observed in supply and demand imbalance may be the result of quality of procurement data as well as underreporting of leishmaniasis cases. Even if most of the countries are still relying on external support in securing access to leishmaniasis products, an increasing number of countries are using domestic funds to procure medicines, and such information was not readily available for this analysis. This may partially explain the smaller supply than demand for MA and MF, which some governments procured through national tenders.

We assumed that the procurement data represent the utilization of medicines for patient treatment in this analysis. However, medicines utilization depends on other factors including national supply chain management, disease programme service delivery, dispensing practice in health facilities and case detection. Therefore, imbalance between supply and demand at national level does not imply that it always led to stock-outs or expiries. Future research should help to better monitor the occurrence of stock-outs and wastage and understand the impact of these incidents on leishmaniasis programme performance and patient treatment outcomes in endemic countries.

In conclusion, this study shows that there are discrepancies in the supply and demand of leishmaniasis medicines at global level and in the top 10 endemic countries. The shortage of medicines or expiration of unused stock due to surplus results in poor programme performance, treatment failures, financial waste and loss of credibility. In order to closely monitor the supply and demand dynamics, coordination among the major procurers and leishmaniasis programme managers is critical. There should be a platform to share procurement planning and case detection rates and generate global forecasts on a regular basis. We also emphasize the importance of manufacturers’ engagement by sharing global forecasts with suppliers, collectively allocating the stock in case of shortages and informing production planning. In addition, efforts to diversify the sources and secure quality-assured, affordable generic products should continue. Antileishmanial medicines are essential life-saving medicines. Leishmaniasis control, hugely underfunded, requires the concerted efforts of partners to consolidate scarce resources and deliver treatments where they are needed by efficient procurement and supply chain management.
